# Impact of rapid investigation clinic on timeliness of lung cancer diagnosis and treatment

**DOI:** 10.1186/s12890-017-0504-5

**Published:** 2017-12-08

**Authors:** Nicole Ezer, Asma Navasakulpong, Kevin Schwartzman, Linda Ofiara, Anne V. Gonzalez

**Affiliations:** 1 Respiratory Epidemiology and Clinical Research Unit, Montreal Chest Institute, McGill University Health Centre, Montreal, QC Canada; 2 Respiratory Division, McGill University Health Centre, Montreal, QC Canada; 3 Respiratory and Respiratory Critical Care Medicine, Faculty of Medicine, Prince of Songkla University, Songkhla, Thailand

**Keywords:** Lung cancer diagnosis, Neoplasm staging, Timeliness, Delay

## Abstract

**Background:**

Guidelines recommend timely evaluation of patients with suspected lung cancer. We evaluated the impact of a Rapid Investigation Clinic (RIC) on timeliness of lung cancer diagnosis and treatment between February 2010 and December 2011.

**Methods:**

Investigation within the RIC was conducted by a pulmonologist and a nurse clinician. Controls were patients with lung cancer, investigated outside the RIC at the same institution during the same time period. The primary outcome was time between first contact with a local physician for suspected lung cancer (T0) and first treatment. Factors associated with the delay from T0 to first treatment were examined using multivariate analysis. Completeness of lung cancer staging according to guidelines was assessed.

**Results:**

A total of 195 patients were investigated within the RIC vs. 132 patients outside the RIC. The median delay between T0 and first treatment was 65 days (interquartile range [IQR] 46–92 days) in the RIC and 78 days (IQR 49–119 days) in the non-RIC patients (*p* ≤ 0.01). Time from T0 to pathological diagnosis was shorter in the RIC (median 26 days; IQR 14–42 days) vs. non-RIC patients (median 40 days; IQR 16–68 days). In multivariate analysis, investigation in the RIC was associated with a reduction in time to first treatment of 24 days (95% confidence interval [CI] 12–35 days) when adjusted for relevant confounders. Guideline-concordant investigation occurred more frequently in RIC patients, based on the quality indicators examined.

**Conclusions:**

A Rapid Investigation Clinic reduces delays to lung cancer diagnosis and treatment, and impacts quality of care.

## Background

Lung cancer remains the leading cause of cancer death for both men and women [1]. Timeliness of lung cancer care is an important quality indicator; however, standards for investigation of new lung cancer patients vary. With the advent of Computed tomography (CT) screening for lung cancer [2] the number of patients requiring investigation is likely to increase. This will require evidence-based strategies aimed at improving guideline-concordant care and minimizing delays.

Accurate lung cancer diagnosis and staging requires various imaging studies and procedures. Positron Emission Tomography (PET) scanning detects unsuspected metastatic disease and reduces non-curative surgical resections [3]. Minimally invasive needle techniques such as endobronchial ultrasound-guided needle aspiration (EBUS) are now considered the test of first choice to confirm mediastinal disease [4]. Work-up of patients with suspected lung cancer requires ready access to these tests and adequate coordination of care, in addition to multi-disciplinary input.

The 2011 National Institute for Health and Clinical Excellence (NICE) guidelines recommend that rapid access clinics should be provided, where possible, for the investigation of patients with suspected lung cancer [5]. The most recent American College of Chest Physicians (ACCP) lung cancer guidelines suggest that efforts be made to deliver “timely” care [6]. At the McGill University Health Centre (MUHC), a Rapid Investigation Clinic (RIC) was established to coordinate and accelerate the workup of patients with suspected lung cancer. The aim of the study was to assess the impact of this model of care on timeliness of lung cancer diagnosis, staging and treatment. The impact of the RIC on the delivery of a guideline-concordant investigation was also examined.

## Methods

The Rapid Investigation Clinic (RIC) was established in February 2010. The RIC operates twice a week, and is staffed by a rotating pulmonary physician and nurse-clinician. The nurse-clinician monitors the investigation progress, assists with coordination of care, and provides patients with the necessary psychosocial support. At initial encounter, preference is given to the invasive diagnostic procedure felt to have the best yield/risk ratio based on CT findings. Procedures that allow simultaneous diagnosis and staging are favored. Patients are staged according to the 2009 staging system [7], and the 2007 American College of Chest Physicians guidelines for lung cancer diagnosis and staging were followed during the study period [8]. Patients in the RIC and non-RIC groups had access to the same imaging studies and diagnostic procedures within the institution.

An institutional database of all patients with a pathological diagnosis of lung cancer was established in November 2008. A prospective database of patients evaluated in the RIC was established in 2010 and maintained by a registrar, to facilitate delays surveillance. Patients investigated within the RIC between February 2010 and December 2011, in whom a diagnosis of lung cancer was confirmed, are included in this analysis (RIC patients). Patients investigated within our institution but outside the RIC, whether by other “non-RIC” pulmonologists or thoracic surgeons, and in whom a diagnosis of lung cancer was confirmed, constituted the comparison group. The “non-RIC” physicians elected to continue investigation of patients with suspected lung cancer within their own clinics, as was being done prior to RIC implementation. Patients with a second lung cancer diagnosis, patients partially investigated outside the institution (e.g. referred only for a specific diagnostic procedure or for surgery), patients undergoing re-staging after initial treatment for lung cancer, and patients investigated during a hospitalization were excluded.

The data extracted from electronic health records included patient demographics, date and type of imaging studies and invasive procedures, histopathological diagnosis, disease stage, date and type of treatments. Performance status was extracted from consultation notes.

Time 0 (T0) refers to the first visit with any MUHC physician for suspected lung cancer, and was defined as follows: 1) In symptomatic patients T0 was the first visit in the ER which triggered further testing for lung cancer; 2) If lung cancer was suspected from imaging, T0 was the visit date after the imaging study, which prompted further investigation; 3) If the patient was followed by a pulmonary physician for an alternate diagnosis or a lung nodule and there was a suspicion of lung cancer, T0 was considered the visit that triggered additional tests; 4) In patients referred to the RIC by any physician, T0 was considered the date of referral. Hence, T0 was not the date of first visit to the RIC, but rather the visit that triggered the lung cancer investigation and/or RIC referral. All delays were measured from T0. The specialty of the physician who evaluated the patient at T0 was recorded.

All invasive diagnostic procedures performed were reviewed. These included bronchoscopy, EBUS (linear or radial), transthoracic needle aspiration (TTNA), endoscopic ultrasound (EUS) and mediastinoscopy. Procedures aimed at sampling a peripheral lung nodule or mass, and procedures aimed at sampling hilar and/or mediastinal nodes were examined separately. The number of non-diagnostic procedures was documented in RIC and non-RIC patients. The invasive diagnostic procedure that first provided a pathological diagnosis of lung cancer was recorded.

The primary outcome was the time interval (in days) between first contact with a local physician for suspected lung cancer (T0) and date of first treatment. First treatment encompassed surgery or radiosurgery for early stage lung cancer, and chemotherapy or combined chemo-radiotherapy for advanced stage disease. The time interval from T0 to date of tissue diagnosis (i.e. date of a pathology report that confirms the diagnosis), and the interval from T0 to the date all staging investigations are completed were also examined. “Staging completed” refers to the date of the last test (imaging study or invasive procedure) that completes the lung cancer investigation according to the ACCP 2007 guidelines [8]. Quality indicators of guideline-concordant investigation were compared in the RIC and non-RIC groups. These included: PET scanning in stage I-II patients treated with surgical resection, brain imaging prior to curative-intent treatment in stage III non-small cell lung cancer (NSCLC), and brain imaging for small cell lung cancer (SCLC). The proportion of patients referred to multidisciplinary lung cancer clinic and/or the lung cancer tumor board was recorded.

Time intervals are reported as median and interquartile range, and were compared using the Wilcoxon rank sum test. Linear regression analysis was used to examine factors thought to be associated with delay from T0 to first treatment. A multivariate model was constructed with the following covariates: age (≤ 75 or >75 years), sex, performance status (Eastern Cooperative Oncology Group (ECOG) 0–1 versus ≥2), lung cancer type (NSCLC versus SCLC), investigation in the RIC versus outside the RIC, inclusion of EBUS and PET scan in the lung cancer investigation, and number of non-diagnostic tests (0, 1 or ≥2). To examine the impact of disease stage, the multivariate analysis was repeated in the subgroup of patients with NSCLC. Quality metrics were compared using a chi-squared test or Fisher’s exact test, as appropriate.

The study was approved by the Research Ethics Board of the MUHC. All analyses were performed using SAS version 9.3. Graphs were designed using GraphPad Prism.

## Results

There were 195 patients in the RIC group and 132 patients in the non-RIC group. Baseline characteristics are detailed in Table 1. The RIC group included a higher proportion of patients with SCLC (13%) compared to the non-RIC group (5%). Performance status was similar in both groups. Among RIC patients, the physician who initiated the lung cancer investigation and referred to the RIC (type of 0 physician) was more likely to be a pulmonologist (93%), while the investigation of non-RIC patients was initiated by both pulmonologists (67%) and thoracic surgeons (27%). During the study period, 103 patients evaluated within the RIC ultimately did not have a diagnosis of lung cancer. These patients were excluded from the analysis. The majority were diagnosed with non-malignant lung pathologies (including sarcoidosis); 14 patients had other primary malignancies; 28 patients did not pursue investigation due to poor performance status; and one patient was diagnosed with a carcinoid tumor.

**Table 1:** Baseline characteristics of patients investigated within the Rapid Investigation Clinic (RIC) and those investigated at the same institution (Non-RIC)

Characteristics	RIC (*N* = 195)	Non-RIC (*N* = 132)	*p*-value^a^
Age in years (mean ± SD)	69 (9)	68 (10)	0.55
Male, N (%)	94 (48)	68 (52)	0.57
NSCLC, N (%)	169 (87)	125 (95)	<0.01
Stage I-II	51 (26)	55 (42)	
Stage III-IV	118 (61)	70 (53)	
SCLC, N (%)	26 (13)	7 (5)	0.29
Extensive Stage	14 (7)	3 (2)	
Limited Stage	12 (6)	4 (3)	
Performance Status, N (%)			0.13
ECOG 0–1	162 (83)	118 (90)	
ECOG 2–3	33 (17)	14 (11)	
Type of T0 Physician, N (%)			<0.01
Pulmonologist	181 (93)	89 (67)	
Thoracic surgeon	–	35 (27)	
Medical oncology	4 (2)	4 (3)	
Internal medicine	6 (3)	–	
Other	4 (2)	4 (3)	

^a^Baseline characteristics were compared using the chi-squared test for categorical variables, and the Student t-test for continuous variables

The number of invasive diagnostic procedures performed was similar in RIC and non-RIC patients (Table 2). The invasive procedures providing the first pathological evidence of lung cancer were directed at lung masses or nodules in 60% of patients in the RIC and 56% of the non-RIC groups (*p* = 0.42). These were directed at lymph nodes in 26% of the RIC and 23% of the non-RIC patients (*p* = 0.22) (Table 3). The last test performed to complete lung cancer diagnosis and staging was most frequently an imaging study. In particular, PET scan was the last test in 48% of RIC and 36% of non-RIC patients (Table 4).

**Table 2:** Number and type of invasive diagnostic procedures performed in RIC versus non-RIC patients

	Patients who underwent a given number of invasive diagnostic procedures (%)	
	RIC (*N* = 195)	Non-RIC (*N* = 132)
Total number of invasive diagnostic procedures performed		
0^a^	9 (5)	10 (8)
1	99 (51)	68 (51)
2	67 (34)	41 (31)
≥3	20 (10)	13 (9)

^a^Includes patients with no invasive procedure performed, and those with pathology only confirmed at the time of surgical resection (wedge resection, lobectomy or pneumonectomy)

**Table 3:** Type of invasive procedures providing the first tissue diagnosis, in RIC versus non-RIC patients

	Number of patients in whom a given type of procedure provided the tissue diagnosis (%)	
	RIC (*N* = 195)	Non-RIC (*N* = 132)
Lung mass or peripheral nodule sampling	**118 (60)**	**74 (56)**
Conventional Bronchoscopy	59 (30)	22 (17)
TTNA	51 (26)	48 (36)
Radial EBUS	8 (4)	4 (3)
Lymph Node sampling	**53 (27)**	**28 (21)**
Linear EBUS	48 (25)	16 (12)
Mediastinoscopy	2 (1)	2 (1)
EUS	0 (0)	4 (3)
Chamberlain	2 (1)	0 (0)
Lymph Node FNA	1 (<1)	6 (<1)
Surgical Sampling^a^	**13 (7)**	**20 (15)**
Biopsy of Metastases^b^	**5 (3)**	**6 (5)**
No pathologic confirmation	**6 (3)**	**4(3)**

^a^At time of lobectomy or wedge resection

^b^Includes thoracentesis, medical thoracoscopy, liver biopsy, brain biopsy, and bone biopsy

**Table 4:** Last imaging test or procedure performed to complete the lung cancer investigation (diagnosis and staging) among patients who received treatment

	Number of patients in whom a given test is the last test to complete the investigation, among treated patients (*N* = 283)	
	RIC (*N* = 167)	Non-RIC (*N* = 116)
Lung mass or peripheral nodule sampling	**12 (7)**	**27 (23)**
Conventional Bronchoscopy	4 (2)	4 (3)
TTNA	7 (4)	23 (20)
Radial EBUS	1 (1)	–
Lymph Node sampling	**26 (16)**	**19 (16)**
Linear EBUS	12 (7)	9 (8)
Mediastinoscopy	10 (6)	8 (7)
EUS	2 (1)	1 (1)
Chamberlain	2 (1)	0 (0)
Lymph Node FNA	1 (<1)	1 (1)
Imaging study	**115 (69)**	**64 (55)**
CT scan	19 (11)	14 (12)
PET scan	80 (48)	42 (36)
MRI	9 (5)	4 (3)
Ultrasound	1 (1)	–
Bone scan	6 (4)	4 (3)
Biopsy of Metastases^a^	**14 (8)**	**6 (5)**
N/A	**1 (−)**	**–**

^a^Includes thoracentesis, medical thoracoscopy, liver biopsy, brain biopsy, and bone biopsy

**Fig. 1:**
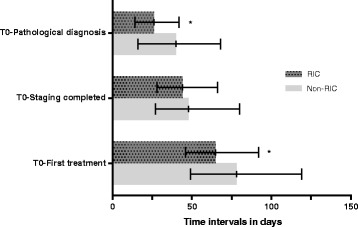
Time intervals from T0 to lung cancer diagnosis, staging and first treatment. * *p* ≤ 0.01

The median delay between T0 and first lung cancer treatment was 65 days (interquartile range [IQR] 46–92 days) for RIC versus 78 days (IQR 49–119) for non-RIC patients (*p* = 0.01). The median delay to first chemotherapy or radiotherapy was significantly reduced in RIC patients (*p* = 0.01), while time to surgery was similar in RIC and non-RIC patients (p = ns). There was no significant difference in time to staging being completed (*p* = 0.39), however time to pathological diagnosis of lung cancer was reduced significantly in the RIC patients (*p* < 0.01) (Fig. 1).

In the multivariate logistic regression analysis, significant predictors of longer intervals to first treatment were being investigated outside the RIC, a pathological diagnosis of small cell lung cancer, age > 75 years, inclusion of PET scan in the work-up, and increasing numbers of non-diagnostic tests (Table 5). Worse performance status and use of EBUS were not associated with longer delays (*p* > 0.05). After adjustment for these variables, investigation within the RIC was associated with a shorter time to treatment (−24 days, 95% confidence interval [CI] -35 to −12 days). Among the subset of patients with NSCLC, patients with stage I –IIB tumors had significantly longer delays to treatment (15 days, 95% CI 3–28) compared to those with later stage tumors when adjusted for investigation within vs. outside RIC, and other confounders.

**Table 5:** Multivariate model of time interval from T0 to first treatment (in days)

All patients (*N* = 283)			Patients with NSCLC only (*N* = 254)		
Characteristic	Beta (days)	*p*-value	Characteristic	Beta (days)	*p*-value
RIC	−24 (−35 to −12)	<0.01	RIC	−24 (−37 to −11)	<0.01
Male	−4 (−15 to 8)	0.53	Male	−2 (−14 to 11)	0.77
Age ≥ 75 years	16 (3 to 30)	0.02	Age ≥ 75 years	14 (0–29)	0.05
ECOG ≥ 2^a^	1 (−16 to 16)	0.98	ECOG ≥ 2^a^	3 (−14 to 11)	0.77
NSCLC (vs. SCLC)	10 (4 to 43)	0.02	Stage I-II (vs. Stage III-IV)	15 (3–28)	0.02
PET	17 (2 to 31)	0.03	PET	13 (−4 to 29)	0.14
EBUS	2 (−11 to 14)	0.20	EBUS	−1 (−14 to 12)	0.90
Number of non-diagnostic procedures			Number of non-diagnostic procedures		
0	Reference	–	0	Reference	–
1	20 (6 to 33)	<0.01	1	18 (5 to 33)	<0.01
≥1	37 (15 to 60)	<0.01	≥1	38 (14 to 60)	<0.01

Patients treated with palliative intent, patients receiving treatment outside the McGill University Health Centre, and patients with time to treatment greater than 1 year were excluded from this analysis. ^a^Reference category is patients with ECOG of 0 or 1

NSCLC; SCLC

Quality indicators were compared in the RIC and non-RIC groups (Table 6). PET scans were performed more frequently in early stage patients investigated within the RIC (94% vs. 82%, *p* = 0.05). Brain imaging in the context of stage IIIA NSCLC was performed in 51% of RIC and 38% of non-RIC patients (*p* = ns). Neuroimaging for SCLC was performed in 92% of RIC and 72% of non-RIC patients (*p* = ns). A larger proportion of patients investigated via the RIC were discussed at tumor board or evaluated in the multidisciplinary lung cancer clinic (74% vs. 55%, *p* < 0.01).

**Table 6:** Pre-specified indicators of guideline-concordant investigation

	RIC (%)	Non-RIC (%)	*p*-value^a^
PET scans in stage I/II NSCLC	48/51 (94)	45/ 55(82)	0.05^a^
Brain imaging in stage IIIA NSCLC	26/ 51(51)	14/ 37(38)	0.22^a^
Brain imaging in SCLC	24/26 (92)	5/7 (72)	0.13^b^
Patient seen in multi-disciplinary lung cancer clinic, or case reviewed at Tumor Board	144/ 195 (74)	73/132 (55)	<0.01^a^

Chi-squared^a^ or Fisher’s exact test^b^ were used

## Discussion

Implementation of a rapid investigation clinic was associated with decreased time between the first visit for suspected lung cancer and first treatment. In multivariate analysis, the difference in time to treatment related to RIC was 24 days, when adjusted for relevant confounders such as sex, age, performance status, inclusion of EBUS and/or PET scan in the investigation, and number of non-diagnostic procedures. The factors associated with significantly longer delays were advanced age, early stage NSCLC, and patients with a first invasive test that was non-diagnostic. Patients investigated within the RIC were more likely to undergo PET scan for early stage lung cancer; they were more likely to be referred to the multidisciplinary lung cancer clinic or have their case reviewed at tumor board. Thus, the rapid investigation clinic improved timeliness of care while improving certain aspects of guideline-concordant care.

The study was based at a single university-affiliated center, which limits the generalizability of the results. The database for RIC patients was maintained prospectively by a trained registrar, with the goal of monitoring investigation delays in real time. In contrast, controls were individuals with a pathological diagnosis of lung cancer identified from the institutional tumor registry, and the investigation path was reviewed retrospectively. However, this definition allowed identification of a comparison group of patients who had access to similar imaging and procedural resources during the study timeframe. The dates and type of tests were easily identified from the institution’s clinical information system, so that delays could be accurately measured and compared. Previous authors have reported that advanced disease may be associated with more prompt investigation and treatment [9, 10]. It is unlikely that sicker patients would be systematically referred to the RIC. In fact, baseline performance status was not significantly different between RIC and non-RIC patients. A larger number of non-RIC patients had early stage NSCLC; however, the RIC-related reduction in delays remained significant after adjustment for stage in the multivariate model of NSCLC patients.

The time required to complete lung cancer staging was comparable in the RIC and non-RIC patients. Several factors may be responsible for lack of change in this regard. EBUS was introduced at our institution at the end of 2008, and was performed by only two operators during the study period. Traditional practice patterns and/or ready availability of certain tests, conventional diagnostic bronchoscopy for example, led to their frequent use in both the RIC and non-RIC patients. In addition, limited access to PET scan delayed completion of staging in both RIC and non-RIC patients. PET scan was the last test needed to complete staging in 48% or RIC vs. 36% of non-RIC patients (Table 4). Despite these challenges, the RIC had a positive impact on time to lung cancer treatment.

Previous studies have evaluated delays to diagnosis or treatment of lung cancer. Olsson et al. systematically reviewed studies describing timeliness of care in patients with lung cancer [11]. Time to diagnosis and treatment of lung cancer were frequently longer than recommended. Salomaa et al. examined delays for 132 patients with NSCLC at a Finnish hospital and reported a median delay from specialist visit to diagnosis of 15 days (mean 55 days), and from diagnosis to treatment of 15 days [12]. More recently a United Kingdom (UK) administrative study of 28,733 patients found that only 43% of patients were treated within 1 month of diagnosis of lung cancer [13]. A United States of America (USA) study of veterans reported median delays of 42 days for diagnosis and 84 days for first treatment [10]. Wait times for diagnosis and treatment in Canada vary across provinces. In Ontario, median time to diagnosis of lung cancer was 37 days (IQR 29–49 days) [14]; in Manitoba median time from abnormal chest x-ray to tissue diagnosis was 26 days, with more than 25% of patients waiting longer than 55 days for diagnosis [15]. In Quebec, median time from initial contact with a physician for lung cancer and surgery was 109 days [16]. The variability in measures is partly due to different definitions of “time 0”.

Guidelines for timeliness of care vary among countries. The 2011 NICE guidelines suggest an acceptable delay is 2 months from urgent general practitioner (GP) referral to beginning of treatment [5]. In this study, the median delay from T0 to first treatment was 81 days, thus not within the recommended 8 weeks. Significant challenges remain in meeting these targets, as highlighted by additional recent studies from Canada and the USA [17–19]. Avoiding diagnostic delays may be increasingly difficult, as accurate staging requires specific procedures and imaging tests [4]. The goal of the RIC was to centralize management of referrals, improve access to specialized providers and diagnostic tests, and standardize the workup of lung cancer. In essence, we attempted to modify both structure and process elements in order to reduce time to treatment.

A systematic review of studies evaluating diagnostic assessment units in patients with solid tumors reported reduced time to first treatment and greater patient satisfaction [20]. When reviewed systematically in patients with lung cancer, factors associated with improved delays included nurse-led coordination of care and use of a “two-stop” investigation pathway, while a multidisciplinary clinic approach did not result in more timely care [11]. Two studies from the UK prospectively implemented a two-stop pathway at centralized hospitals with a team dedicated to investigation and staging; both decreased time to diagnosis and increased radical treatment rates by scheduling patients for procedures the same day as physician evaluations [21, 22]. A single-center, Veterans’ Administration (VA) study from the USA retrospectively reviewed timeliness of care provided in a multidisciplinary clinic with “usual” care provided after the clinic closed. The comparison revealed similar intervals to diagnosis and treatment with a multidisciplinary approach; the authors hypothesized this may have been due in part to absence of a surgeon in the multidisciplinary setting, and existing infrastructure from the previous multidisciplinary clinic [23]. A VA study in Birmingham reported improved lung cancer resection rates after implementation of a “Lung Mass Clinic” staffed by specialists in lung cancer, although the median time to resection was longer than expected (104 days) [24]. These conflicting results highlight the complexity of lung cancer care. Medical complexity was shown to increase delays to treatment in a recent Norwegian study, yet too few of even the least complex patients received timely treatment [25].

The impact of timeliness of care on lung cancer outcomes is unclear. In the systematic review of Olsson et al., the association between timely lung cancer care and patient outcomes were mixed and, at times, paradoxical [11]. A large population-based study of surgical patients demonstrated no influence of time to surgery on survival [26]. A Swedish study of 466 patients treated with curative or palliative intent showed that those with shortest time to treatment had worse survival [27]. Gould et al. investigated 129 veterans with NSCLC and found that more timely care was not associated with better survival. More advanced patients may require more urgent evaluation, thereby decreasing overall survival in patients treated sooner. However, in the subgroup of patients with solitary pulmonary nodules, there was a trend toward improved survival with shorter time to treatment [10]. O’Rourke et al. reported tumor growth while patients waited for radiotherapy treatment by comparing diagnostic and CT simulation scans, suggesting that delays to treatment may be associated with worse outcomes [28].

No study may be sufficiently powered to assess the survival benefit of a rapid access clinic model, and efforts to improve more scalable quality outcomes may be more realistic. Quality gaps have been defined as differences between health-care processes observed in clinical practice and those recommended by evidence-based guidelines [29]. Ost and colleagues examined quality gaps in lung cancer diagnosis and staging, using the Surveillance Epidemiology and End Results (SEER) database and the Texas Cancer Registry. Patients with lung cancer with regional spread and no distant metastases were classified as receiving guideline-consistent care if they underwent mediastinal lymph node sampling as the first invasive test; only 21% of patients had guideline-consistent diagnostic evaluations [30]. In the current study, implementation of a rapid access clinic was associated with more frequent guideline-concordant care, based on the quality indicators examined.

## Conclusion

The implementation of a rapid investigation clinic for patients with lung cancer reduced delays to diagnosis and treatment and improved quality of care. Continued monitoring of investigation pathways and wait times is necessary to ensure barriers and quality gaps are addressed in a timely manner.

## Abbreviations

ACCP: American College of Chest Physicians
CI: Confidence interval
CT: Computed tomography
EBUS: Endobronchial ultrasound
ECOG: Eastern Cooperative Oncology Group
EUS: Endoscopic ultrasound
GP: General Practitioner
IQR: Interquartile range
MUHC: McGill University Health Centre
NICE: National Institute for Health and Clinical Excellence
NSCLC: Non-small cell lung cancer
PET: Positron emission tomography
RIC: Rapid Investigation Clinic
SCLC: Small cell lung cancer
SEER: Surveillance Epidemiology and End Results
TTNA: Transthoracic needle aspiration
USA: United States of America
UK: United Kingdom
VA: Veterans’ Administration
